# Efficacy and Safety of Low-Dose Pemafibrate Therapy for Hypertriglyceridemia in Patients with Type 2 Diabetes

**DOI:** 10.31662/jmaj.2020-0104

**Published:** 2021-03-26

**Authors:** Hidenori Bando, Shinji Taneda, Naoki Manda

**Affiliations:** 1 Manda Memorial Hospital, Sapporo City, Hokkaido, Japan

**Keywords:** pemafibrate, triglyceride, hypertriglyceridemia, type 2 diabetes

## Abstract

**Introduction:**

Pemafibrate is a potent selective peroxisome proliferator-activated receptor α (PPARα) modulator that may be safer than conventional PPARα agonists in the treatment of dyslipidemia. This study was designed to investigate the efficacy of low-dose pemafibrate (0.1 mg/day) therapy for hypertriglyceridemia in 31 patients with type 2 diabetes and high triglyceride (TG) levels at the Manda Memorial Hospital.

**Methods:**

TG, remnant lipoprotein cholesterol (RLP-C), high-density lipoprotein cholesterol (HDL-C), low-density lipoprotein cholesterol (LDL-C), apolipoprotein (Apo) AI, Apo AII, Apo B, Apo CII, Apo CIII, and Apo E levels were evaluated. Liver, kidney, and muscle toxicity tests were also performed. Pemafibrate (0.1 mg) was administered once daily.

**Results:**

This treatment significantly decreased TG, RLP-C, Apo CII, Apo CIII, and Apo E levels while significantly increasing HDL-C, Apo AI, and Apo AII levels. No significant changes were observed in LDL-C and Apo B levels. There were no significant liver-, kidney-, or muscle-related adverse events.

**Conclusions:**

The results of this study show that low-dose pemafibrate administration improves the lipid profile in Japanese patients with hypertriglyceridemia and type 2 diabetes.

## Introduction

Lifestyle-related diseases, such as diabetes mellitus, hypertension, stress, and dyslipidemia, as well as habitual smoking, are risk factors for atherosclerosis and cardiovascular events ^(1)^. A reduction in the levels of low-density lipoprotein cholesterol (LDL-C) can prevent the development of cardiovascular diseases and atherosclerosis. However, even when LDL-C levels are reduced by drug therapies, such as statin treatment, there is a residual risk of cardiovascular diseases ^(2), (3)^. Thus, additional therapies are required to reduce the risks associated with high triglyceride (TG) levels. Peroxisome proliferator-activated receptor α (PPARα) agonists are potential candidates for this type of additional therapy ^(4)^. Pemafibrate is a potent selective PPARα modulator that has a favorable benefit-risk balance. It may also be safer than conventional PPARα agonists ^(5), (6)^. Clinically, pemafibrate can be effectively and safely administered for decreasing TG levels while reducing the incidence of abnormal liver and renal function parameters compared with conventional PPARα agonists ^(7), (8)^. However, in contrast to conventional agents, which are principally excreted via the kidneys, pemafibrate is primarily excreted via the liver ^(9)^. Some patients show poor adherence to PPARα agonists, and some cannot tolerate them at the normal dose because of associated adverse effects (AE). Although the normal dose of pemafibrate is 0.2 mg, in clinical practice, 0.1 mg/day pemafibrate can decrease TG levels and increase high-density lipoprotein cholesterol (HDL-C) levels ^(10)^; however, this effect has not been examined in patients with type 2 diabetes. In the present study, the efficacy and safety of 0.1 mg/day pemafibrate in patients with type 2 diabetes and dyslipidemia in clinical practice was reported.

## Materials and Methods

### Trial design

This study was a single center, retrospective study conducted at the Manda Memorial Hospital. Patients with type 2 diabetes mellitus and hypertriglyceridemia who had repeated high TG were enrolled. Treatment with 0.1 mg pemafibrate once daily was started in 2019 and continued for 3 months to evaluate its efficacy. Blood levels of the following lipids were determined: TGs, remnant lipoprotein cholesterol (RLP-C), LDL-C, HDL-C, non-HDL-C, apolipoprotein (Apo) AI, Apo AII, Apo B, Apo CII, Apo CIII, and Apo E. Hemoglobin A1c (HbA1c) and plasma glucose (PG) levels were also measured to evaluate the status of glycemic control in these patients with type 2 diabetes. To evaluate drug safety, the following blood tests for liver function were performed: serum aspartate aminotransferase (AST), alanine aminotransferase (ALT), and γ-glutamyl transpeptidase (γ-GTP). To assess renal function, serum creatinine (sCr) and the estimated glomerular filtration rate (eGFR) were determined. Serum creatine kinase (CK) levels were also evaluated to identify rhabdomyolysis and myopathy. Patients with missing data at baseline or at the end of the study were excluded. However, three patients who were only missing sCr and eGFR data were included. Moreover, two patients who had previously taken other fibrates were switched to 0.1 mg pemafibrate for the duration of the three-month study. A total of 31 patients were ultimately enrolled.

### Statistical analyses

A paired *t*-test was performed to compare laboratory data before and after 0.1 mg/day pemafibrate administration for 3 months. Statistical analyses were conducted using EZR ^(11)^ with a significance level of 5% and a two-sided confidence coefficient of 95%. Differences with a p-value of less than 0.05 indicated significance. Data are presented as mean ± standard deviation.

### IRB approval number and name of the institution

All procedures were in accordance with the ethical standards of the responsible committee on human experimentation (institutional and national) and with the Helsinki Declaration of 1964 and later versions. This study was approved by the ethics committee of the Manda Memorial Hospital (approval number, 2020-5; approval date, June 19, 2020). Although informed consent was not obtained from the participants, they were provided with the opportunity to deny participation by posting the opt-out document.

## Results

Table 1 shows the demographics and clinical characteristics of patients at baseline. A total of 31 patients were enrolled in this study and administered 0.1 mg/day pemafibrate for 3 months. Regarding the timing of blood sampling, at baseline and at the end of the study, 5 patients were fasting and 26 were postprandial. Table 2 presents the various antidiabetic agents used by patients.

**Table 1:** Characteristics of Patients.

Age, years		62.0 ± 13.2
Sex, male, n		22
Fasting/postprandial^1^, n		5/26
Body weight, kg		74.7 ± 14.1
BMI kg/m^2^		28.00 ± 4.45
Duration of DM, years		12.3 ± 6.83
Alcohol (above 140 g/week), n		8
Fibrate, n		2
Statin, n		14
Ezetimibe, n		7
EPA/DHA^2^, n		1

Age, body weight, BMI, duration of DM: average ± standard deviation (SD) ^1^The timing of blood sampling: fasting/postprandial ^2^EPA/DHA: eicosapentaenoic acid/Docosahexaenoic acid.

**Table 2.** Antidiabetic Agents.

Insulin, n		8
GLP-1RA, n		6
DPP4I, n		13
SGLT2I, n		14
BG, n		12
SU, n		5
αGI, n		4
TZD, n		1
Glinide, n		6

GLP1RA, GLP1 receptor agonist; DPP4I, DPP4 inhibitor; SGLT2I, sodium-glucose transporter 2 inhibitor; BG, biguanide; SU, sulfonylurea; αGI, α-glucosidase inhibitor; TZD, thiazolidine derivative.

### Efficacy of the treatment

Table 3 shows changes in the lipid and lipoprotein levels after the treatment. After 3 months of pemafibrate treatment, TG and RLP-C levels of patients significantly decreased (p < 0.001) and HDL-C levels significantly increased (p = 0.001). Changes in non-HDL-C and LDL-C levels from baseline to the end of the study were not significant (p = 0.054 and 0.52, respectively). Table 4 shows the efficacy of pemafibrate in terms of Apo levels. Apo AI and Apo AII levels were significantly higher (p < 0.001) at the end of the study than at baseline. The change in Apo B level was not significant (p = 0.39); however, Apo CII, Apo CIII, and Apo E levels significantly decreased (p = 0.02, 0.004, and 0.007, respectively).

**Table 3.** Efficacy Profile 1.

	Before	At the end	p-value	Mean of differences	95% CI
TG (mg/dl)	296.9 ± 126.0	205.7 ± 84.7	0.001>	90.6	47.2 to 134.1
RLP-C	10.6 ± 5.28	6.53 ± 3.17	0.0001>	4.06	2.39 to 5.73
HDL-C	46.4 ± 10.0	51.6 ± 10.3	0.001	−5.19	−8.12 to 2.27
Non-HDL-C	148.5 ± 26.3	140.2 ± 32.7	0.054	8.23	−0.14 to 16.6
LDL-C	103.0 ± 26.3	106.1 ± 28.7	0.52	−3.1	−12.8 to 6.62

Comparison of lipid values before and 3 months after (at the end) the administration of pemafibrate Paired *t*-test before, at the end: average ± standard deviation (SD) confidence interval (CI).

**Table 4.** Efficacy Profile 2.

	Before	At the end	p-value	Mean of differences	95% CI
Apo A1	130.8 ± 18.8	139.1 ± 18.7	0.0000303	−8.23	−11.6 to 4.80
Apo A2	32.3 ± 5.8	38.8 ± 6.80	0.00001>	−6.53	−8.96 to 4.09
Apo B	89.9 ± 13.6	87.9 ± 16.8	0.40	2.03	−2.72 to 6.79
Apo C2	8.62 ± 2.03	7.81 ± 2.21	0.02	0.81	0.13 to 1.48
Apo C3	18.3 ± 6.20	15.5 ± 5.25	0.004	2.78	0.97 to 4.59
Apo E	3.55 ± 1.33	3.01 ± 1.04	0.007	0.54	0.16 to 0.91

Comparison of apolipoprotein values before and 3 months after (at the end) the administration of pemafibrate Paired *t*-test before, at the end: average ± standard deviation (SD) confidence interval (CI)

### Safety

Table 5 shows the safety data. The liver function assays showed that AST levels did not significantly change after 3 months of 0.1 mg/day pemafibrate (p = 0.171). ALT and γ-GTP levels were lower at the end of treatment compared with the baseline (p = 0.026 and 0.004, respectively). There was no significant change in the CK level after 3 months of treatment (p = 0.962). In terms of renal function, there were no significant changes in the sCr and eGFR levels after 3 months of treatment (p = 0.95 and 0.874, respectively = 28). Moreover, the HbA1c level increased by 0.22% (p = 0.002), while the PG level was not significantly affected (p = 0.819). When the timing of blood sampling was the same, no significant change in blood glucose levels was observed.

**Table 5.** Safety Profile.

	Before	At the end	p-value	Mean of differences	95% CI
AST	28.4 ± 14.1	26.7 ± 10.6	0.171	1.61	−0.73 to 3.96
ALT	35.1 ± 27.0	29.1 ± 18.2	0.0261	6.06	0.77 to 11.35
γ-GT	70.4 ± 77.0	51.9 ± 55.3	0.00424	18.5	6.30 to 30.74
CK	118.2 ± 70.2	117.7 ± 67.9	0.962	0.48	−20.2 to 21.2
Cr	0.85 ± 0.17	0.86 ± 0.18	0.95	−0.001	−0.04 to 0.03
eGFR	67.2 ± 14.0	67.2 ± 13.4	0.874	0.185	−2.20 to 2.57
HbA1c	7.46 ± 1.03	7.68 ± 0.98	0.002	−0.22	−0.36 to 0.09
PG	172.1 ± 57.9	170.2 ± 48.8	0.819	1.9	−15.0 to 18.8

Comparison of laboratory data values before and 3 months after (at the end) pemafibrate administration Paired *t*-test (Cr and eGFR, n = 28) Before, at the end: average ± standard deviation (SD) CI, confidence interval.

## Discussion

The normal dose of pemafibrate is 0.2 mg (0.1 mg administered twice a day). Most previous studies have followed this schedule. However, in clinical practice, there is doubt whether 0.1 mg twice a day should be used in most cases. In a phase 2 Japanese study ^(12)^, the efficacy and safety of 0.1 mg/day pemafibrate (administered as 0.05 mg twice daily) vs. 0.2 mg/day (administered as 0.1 mg twice daily) was compared. The clinical effect of pemafibrate on TGs was greater when administered at 0.2 mg/day than that at 0.1 mg/day, and its effect on HDL-C was similar at both doses. In contrast, AE and adverse drug reactions (ADR) were fewer at 0.1 mg/day than at 0.2 mg/day. In a phase 3 randomized placebo-controlled Japanese study ^(7)^, the clinical effects of pemafibrate on TG, HDL-C, and RLP-C levels were weaker when administered at 0.1 mg/day than at 0.2 mg/day, and the percentages of AE and ADR were lower at 0.1 mg/day than at 0.2 mg/day. In a systematic review of studies that used pemafibrate at a dose of 0.1 mg/day, the effect of the drug was more favorable than that of placebo and as good as fenofibrate in terms of its effect on TGs ^(13)^. Taken together, these findings revealed that the use of 0.1 mg/day pemafibrate for dyslipidemia is effective and safe, particularly as it improves TG, HDL-C, and RLP-C levels, thus reducing the residual risk of atherosclerosis. The results of the present study support the use of pemafibrate at a dose of 0.1 mg/day. Considering that adherence was higher with this once-daily regimen than with twice-daily treatment, low-dose (0.1 mg/day) pemafibrate is useful and safe for patients with dyslipidemia. In clinical practice, 0.1 mg/day pemafibrate administration was effective in significantly reducing TG and elevating HDL-C levels. In a previous study ^(10)^, this regimen was shown to improve dyslipidemia in patients without diabetes. The present study, in which only patients with type 2 diabetes were included, suggests that this regimen is safe and effective in treating dyslipidemia.

Pemafibrate is also known to affect Apo ^(7), (12)^. Apo E exists in chylomicrons, intermediate-density lipoproteins, and very-low-density lipoproteins ^(14)^. In addition, Apo CII, Apo CIII, and Apo E play important roles in the metabolism and clearance of TG-rich lipoproteins ^(14), (15), (16)^. In the present study, the levels of these Apo were decreased by 0.1 mg/day pemafibrate administration. In fact, the levels of Apo AI and Apo AII, which are present in HDL-C, significantly increased. The former constitutes almost all of HDL-C and the latter two-thirds of HDL-C ^(17)^. Following the administration of 0.1 mg/day pemafibrate, in contrast to Apo AI and Apo AII levels, Apo CII and Apo CIII levels decreased. Apo E was not measured ^(7), (12)^. The increase in Apo AI and Apo AII levels and decrease in Apo CII, Apo CIII, and Apo E levels indicate that Apo associated with TG and HDL-C were favorably affected by 0.1 mg/day pemafibrate. Moreover, AST levels significantly increased at 10 and 12 weeks after the administration of 0.1 mg/day pemafibrate. In contrast, ALT and γ-GTP levels were significantly lower at 4-12 weeks ^(7)^. However, CK, sCr, and eGFR levels did not change ^(7)^. In the current study, although lipid levels were improved in patients with diabetes, HbA1c levels significantly increased for unclear reasons. In the PROVIDE study, patients were administered pemafibrate (0.2 or 0.4 mg/day), and their HbA1c level increased ^(18)^. The HbA1c level might be affected by time-dependent changes observed in patients with diabetes, or possibly, this increase was not related to glucose changes. Fibrates improve red blood cell deformability by modifying erythrocyte membrane lipids ^(19)^, which might affect erythrocyte dynamics and lifespan, as well as the HbA1c level. In a model of insulin resistance, pemafibrate decreased fasting PG and insulin levels and disrupted homeostasis ^(20), (21)^. In the present study, the glucose level was not significantly altered when the fasting and postprandial patients were combined. Thus, although this is not a sufficient assessment of insulin resistance, it is believed that the lack of concordance between glycemic variability and HbA1c changes was adequately assessed under pemafibrate use. Moreover, in the previous study, habitual alcohol drinkers were excluded; however, in the present study, these individuals were included, and the results show that pemafibrate was effective in treating dyslipidemia, particularly in patients with high TG and low HDL-C levels, regardless of whether alcohol was consumed habitually or not.

Considering that the present study was performed in a single center, the laboratory analyses and conditions were stable and fixed. Nevertheless, there were certain limitations noted in the study. First, the laboratory data included results for fasting and postprandial patients; thus, the results did not exclude the effect of food intake. Second, the study period was only 3 months. This was insufficient to evaluate the long-term efficacy of low-dose pemafibrate for treating dyslipidemia, particularly in terms of atherosclerotic risk. To evaluate its long-term efficacy, a longer period of administration is needed. Finally, only patients with type 2 diabetes were included in this study. Therefore, the results do not apply for patients without diabetes or with type 1 diabetes. Diabetes has been considered in previous phase 2 and 3 studies, and the effect of 0.1 mg/day pemafibrate in patients without diabetes has been described elsewhere.

### Conclusions

In patients with dyslipidemia and type 2 diabetes, 0.1 mg/day pemafibrate decreased the levels of TG and RLP-C and increased the levels of Apo involved in TG and HDL-C metabolism, thereby increasing HDL-C levels. The favorable safety profile of pemafibrate, including the lack of AE, as evidenced by kidney- and liver-related laboratory data, support the efficacy of low-dose pemafibrate (0.1 mg/day) as a treatment option for patients with diabetes.

## Article Information

### Conflicts of Interest

Taneda S. received honoraria for lectures from Takeda Pharmaceutical Co., Ltd., and Novo Nordisk Pharma.

### Acknowledgement

We would like to thank Editage (www.editage.com) for English language editing.

### Author Contributions

The corresponding author and coauthors contributed to the following four criteria:

1. Substantial contributions to the conception or design of the work or the acquisition, analysis, or interpretation of data for the work;

2. Drafting the work or revising it critically for important intellectual content;

3. Final approval of the version to be published;

4. Agreement to be accountable for all aspects of the work in ensuring that questions related to the accuracy or integrity of any part of the work are appropriately investigated and resolved.

### Approval by Institutional Review Board (IRB)

All procedures followed were in accordance with the ethical standards of the responsible committee on human experimentation (institutional and national) and with the Helsinki Declaration of 1964 and later versions. This study was approved by the ethics committee of the Manda Memorial Hospital (approval number, 2020-5; approval date, June 19, 2020). Although informed consent was not obtained from the participants, they were provided with the opportunity to deny participation by posting the opt-out document.
